# Effect of the Kangaroo Mother Method after Preterm Delivery on Maternal Stress and Anxiety in the Context of the COVID-19 Pandemic—A Cohort Study

**DOI:** 10.3390/ijerph192416432

**Published:** 2022-12-07

**Authors:** Delia Cristóbal-Cañadas, Tesifón Parrón-Carreño, Bruno José Nievas-Soriano

**Affiliations:** 1 Neonatal and Pediatric Intensive Care Unit, Torrecárdenas University Hospital, 04009 Almeria, Spain; 2 Nursing, Physiotherapy and Medicine Department, University of Almeria, 04120 Almeria, Spain; 3 Andalusian Council of Health at Almeria Province, 04005 Almeria, Spain

**Keywords:** kangaroo mother method, neonatal intensive care unit, stress, anxiety

## Abstract

Background: The kangaroo mother method (KMM) may benefit infants and mothers in many ways. However, few studies focused on its efficacy on maternal anxiety and stress, especially in the context of the COVID-19 pandemic. Objective: To examine the effect of the kangaroo mother method (KMM) on postnatal stress and anxiety in mothers of preterm infants in neonatal intensive care, in the context of the COVID-19 pandemic. Methods: A cohort study of two groups of 56 mother-infant dyads recruited from a neonatal intensive care unit was conducted. Two groups were compared in terms of the mean duration of KMM during the twelve days of the study: the intervention group (mean duration of more than ninety minutes per day) and the control group (less than ninety minutes). Maternal stress was measured using the Parental Stressor: Neonatal Intensive Care Unit (PSS: NICU) scale and STAI E/R questionnaire before and after intervention (KMM). Demographic and other maternal covariates were extracted from medical records. Daily NICU records were used to track the frequency and duration of KMM sessions. Results: Mothers of the intervention group scored lower on the PSS: NICU and STAI E/R, although no statistically significant differences were found (*p* > 0.05). Conclusions: Contrary to research based on biological and physiological parameters in newborns or performed before the COVID-19 pandemic, the differences found in applying subjective scales in mothers in the context of the COVID-19 pandemic were not significant. Therefore, mothers’ perception of physical contact with their preterm infants may not have been as positive due to their fear of transmitting COVID.

## 1. Introduction

The impact of stress on parents of very preterm infants in the neonatal intensive care unit (NICU) is well documented [1]. Parents of preterm infants are affected by several stressors inherent to the NICU, such as sights and sounds, infant behavior and appearance, altered parental functions, and staff behavior [2]. Separating the preterm infant from the parents and disrupting the mother–infant relationship are essential stressors [3]. Research shows that maternal stress caused by separation does not diminish over time; mothers of preterm infants may experience stress and parenting disorders that persist beyond the NICU [4]. Maternal stress in the newborn and later in life is also associated with later infant behavioral and learning problems [5]. Although increased maternal stress and post-traumatic stress disorder are well documented in mothers of late preterm infants [6], little is known about mothers of very preterm or moderate preterm infants. Available data suggest that mothers who deliver preterm are more likely to have difficulty breastfeeding after hospital discharge and may be at increased risk for postpartum depression and post-traumatic stress disorder [5].

The World Health Organization (WHO) defines the kangaroo mother method as “the care of premature infants carried skin-to-skin with the mother. It is a safe, effective, and easy-to-use method to promote the health and well-being of infants born prematurely and at term.” The definition includes as key features “early, continuous and prolonged skin-to-skin contact between mother and baby, exclusive breastfeeding (ideally) and early discharge from the hospital” [7]. Various forms of KMC have been adopted worldwide [8], depending on the needs of different settings. This diversity includes exclusive and non-exclusive breastfeeding, breast or tube feeding, full or partial nudity, continuous (≥20 h per day) or intermittent (short periods of one or more times per day and for a variable number of days) skin-to-skin contact with varying exposure times, and early or non-early hospital discharge [9]. In resource-rich countries, skin-to-skin contact is considered an adjunct to incubator care, so continuous skin-to-skin contact is rare. The WHO also reported that kangaroo care is the most effective method for maintaining body temperature, preventing infection, stimulating the senses, and providing maternal love for the baby’s well-being [7]. In addition, kangaroo care is beneficial for premature infants because it helps maintain regular breathing and reduces energy consumption, which allows infants to gain the weight they need [9,10]. World Health Organization (WHO) guidelines [7] recommend initiating kangaroo mother care after stabilization. A recent multicenter study in five tertiary hospitals demonstrated that initiation of continuous kangaroo mother care soon after birth for infants with birth weights between 1.0 and <1.8 kg improved neonatal survival by 25%, compared to kangaroo mother care initiated after stabilization. According to some authors, the results could be generalizable to hospitals in low-resource settings [7,11].

Postpartum anxiety and stress in women are influenced by many factors, the main ones being the child’s condition, the family environment, and the mother’s characteristics [12]. Among them, the clinical condition of preterm infants is perceived as an essential risk factor for anxiety and stress. In this regard, Treyvaud et al. [13] reported that stress and anxiety levels were significantly increased in mothers of preterm infants compared to full-term infants.

Maternal contact can buffer infant stress, whereas deprivation increases stress sensitivity [14]. Skin-to-skin contact between mother and child reduces maternal symptoms of postpartum depression and improves mother-infant bonding [15,16,17]. When the baby is born prematurely, women give birth to children for whom they are not mentally prepared [18]. In addition, preterm birth and the subsequent hospitalization of newborns in a NICU involve an extraordinary life situation for women in which the maternal role begins and evolves in a public and medical context [19]. Other stressors include doubts about the baby’s survival, the family’s ability to care for the baby, feelings of guilt, and physical and psychosocial concerns [20]. Mothers are often the primary caregivers of their infants and have to cope with care needs that are constant and complex. Thus, stress can affect parental communication and behavior, which impacts parents’ relationships and interactions with their children. Stress has also been linked to behavioral problems in children and can affect optimal parenting [21]. Maternal mental health symptoms have negatively influenced the mother-child relationship and attachment [22,23]. So far, studies have been published evaluating the efficacy of an intervention to help alleviate distress in parents of preterm infants. However, few studies have explicitly focused on maternal post-traumatic stress symptoms [24,25]. A previous study on how kangaroo care would influence the stress of preterm infants estimated the effect of the kangaroo mother method (KMM) on physiological and biochemical parameters and found statistically significant differences in favor of the group that spent more time in kangaroo care [26]. Therefore, this study aimed to evaluate the effect of the mother kangaroo method on mothers of preterm infants and its influence on maternal stress and anxiety in the first days of neonatal intensive care.

## 2. Materials and Methods

### 2.1. Study Design and Participants

A prospective cohort study was conducted in the Neonatal Intensive Care Unit (NICU) of the Torrecardenas Mother and Child Hospital, the reference hospital for the province of Almeria, located in the southeast of Spain. Mothers with newborns aged 28–34 weeks gestation and admitted to the NICU were included in the study. Mothers signed informed consent before the start of the study.

### 2.2. Study Procedures and Outcome Variables

Mothers who agreed to participate in the study were required to perform kangaroo mother method sessions. For ethical reasons, a purely random assignment was not possible. Depending on the stability of the newborn or the mother’s availability to perform the kangaroo method, they were subsequently divided into two groups. The KMM sessions consisted of placing the newborn, dressed only in diapers, in direct contact with the mother’s skin, head to one side, legs and arms flexed, with the mother holding her breast. The mother was reclined in a chair with a blanket over her chest. KMM sessions lasted at least ninety minutes per day. The WHO [7] recommends avoiding kangaroo sessions of less than 60 min. Systematic reviews and other recent research [1,27] have estimated the benefits of this method to be between 60 and 120 min. This average time has been used as a reference. Once the twelve days of the study had passed, the mothers were retrospectively divided into two groups, the intervention group (*n* = 56) being those who had spent an average of ninety minutes a day during the study period and the control group (*n* = 56) those who had spent an average of less than this time. The duration of each KMM session was determined by the infants’ clinical condition and the mothers’ willingness. All preterm infants, with or without kangaroo care, received usual neonatal care according to NICU protocols. Parents could visit their infants at any time.

The outcome variables were: the mother’s demographic data and information and the child’s anthropometric data (age, type of delivery, gestational age, birth weight, The variables analyzed were two previously validated instruments: Parental Stress Scale: Neonatal Intensive Care Unit (PSS: NICU), and the State-Trait Anxiety Questionnaire (STAI A-R). The scales were recorded first in a baseline measurement on the third day of life, the day the kangaroo mother program was initiated (pre-intervention). The second data collection was performed twelve days after the start of the kangaroo program (post-intervention). A triple-blinded analysis was performed.

Maternal stress and anxiety were measured using the Parental Stress Scale (PSS: NICU) and State-Trait Anxiety Questionnaire (STAI A-R) questionnaires.

The Parental Stress Scale: Intensive Care Unit (PSS: NICU) measured the level of parental stress in Neonatal Intensive Care. It was developed and validated by Miles et al. [28] to measure parental stress resulting from the physical and psychosocial environment of a Neonatal Intensive Care Unit. To measure the instrument’s reliability, they used Cronbach’s Alpha correlation coefficient, resulting in 0.96. It was also validated in Latin America by Ruiz et al. [29] and Jofré et al. [30] with a Cronbach’s Alpha of 0.87, showing high inter-item reliability. The scale consists of a questionnaire with 26 closed-ended items, with 5-point Likert-type answers ranging from 1 (not at all stressful) to 5 (extremely stressful). The scale established the parents’ general level of stress and experiences that they had experienced as stressful when their child was admitted to intensive care. Parents who had not experienced a particular situation in an item indicated it with the “not relevant” response.

The PSS: NICU questionnaire was translated from English to Spanish by two native Spanish speakers fluent in English and then translated into English by an independent translator to provide quality control of the translation [31]. The questionnaire included three dimensions: images and sounds of the unit (6 items), infant behavior and appearance (13 items), and relationship with the infant and her role as a mother (7 items). A score was obtained for each subscale. A total score for each mother was obtained by adding all the scores for each question within each subscale.

According to Miles [28], the PSS: NICU scale can be scored in two ways: Metric 1, the level of stress occurrence, is the level of stress experienced in a given situation. Those who reported the experience received a score and those who did not were coded as not found. The total denominator is the number of parents who experienced stress. Metric 2, the overall stress level, is the stress of the entire NICU environment. Mothers who did not report a score on an item received a score of 1. The total denominator is the number of items in the scale.

The State-Trait Anxiety Inventory (STAI E/R) [32], adapted for the Spanish population by TEA 1982, was used to evaluate anxiety. It has two subscales that can be used independently and that measure two dimensions of anxiety: State-Trait Anxiety (STAI-E) and Trait Anxiety (STAI-R). The authors define state anxiety as a transient emotional condition of the organism, characterized by subjective feelings of tension and apprehension. Trait anxiety is a stable propensity to perceive people and situations as threatening, thus raising anxiety. This tool consists of 40 items (20 for each subscale), and the rating is recorded according to sex and age. The results can be expressed according to percentiles, decatypes, or anxiety levels. The questionnaire has an excellent internal consistency in Spanish adaptation, between 0.9 and 0.93 in anxiety/state and between 0.84 and 0.87 in anxiety/trait [32].

### 2.3. Sample Size Estimation

Sample size calculation was performed based on previous studies [33,34] and based on the results of the PSS: NICU questionnaire, using the software Epidat 4.2 (General Directorate of Public Health of the Junta de Galicia, Spain). The objective was to detect mean differences of 0.74 with expected standard deviations of 0.97 for the first group and 1.05 for the second group. The power was 80%, and a confidence level of 95%. An estimated sample size of 31 individuals in each group was obtained.

### 2.4. Statistical Analyses

Data were analyzed with the SPSS statistical package (IBM SPSS, Armonk, NY, USA) version 27.0. Frequencies and percentages were used for qualitative variables and means and standard deviation were calculated for quantitative variables. The Chi-Square test was used to compare qualitative variables. Student’s *t*-test and Mann-Whitney U test were used to compare the means of quantitative variables. The Kolmogorov-Smirnov test was used to determine normality. Pearson’s correlation (r) was used to correlate normal data, and Spearman’s correlation coefficient (rs) when any of the variables did not follow a normal distribution. Statistical significance was considered when *p* < 0.05.

### 2.5. Ethical Aspects and Review Board Approval

The study was conducted under the statements of the Declaration of Helsinki. Informed consent was obtained before taking part in the research. The confidentiality of the participants was guaranteed, and no personal data were collected. This study was approved by the Research and Ethics Committee of the Torrecardenas University Hospital of Almeria, with approval reference PI.DCC/MMC-2019.

## 3. Results

### 3.1. Demographic and Clinical Features

One hundred and twelve mothers and preterm infants participated in the study. They were randomly distributed between the two groups, the intervention group and the control group, with 56 participants. The mean daily duration of kangaroo care was 127.7 min, with an SD of 30.1 min in the kangaroo group. In the no kangaroo group, the mean daily duration was 83.2 min, with an SD of 29.9 min (*p* < 0.001, Student’s *t*-test). The median duration of the kangaroo method before splitting was 88.8 min. After splitting, it was 121.3 min for the intervention group and 40.0 min for the control group. Their main demographic and clinical characteristics are shown in Table 1. The demographic and clinical features of the mothers at the time of the intervention, gestational age (weeks), and the birth weight of the preterm infants were homogeneous in both study groups (intervention and control).

### 3.2. Questionnaires Applied

The mean scores of the PSS: NICU questionnaire were slightly higher in the KMM group, although no statistically significant differences were found (Table 2).

After applying the KMM (post-intervention, day fifteen), mean scores on the PSS: NICU and STAI A/R questionnaires were lower in the intervention group. However, no statistically significant differences were found (*p* > 0.05).

The scores of the PSS: NICU, and STAI S/T scales pre- and post-intervention were analyzed regarding their correlation with maternal age. No significant correlations were found between the latter variable and any of them.

## 4. Discussion

The main objective of the present study was to examine the effect of the kangaroo mother method and its influence on maternal stress and anxiety in the first days of neonatal intensive care. Other studies have found that the KMM may play a role in regulating negative emotions such as depression and stress in mothers after childbirth [35], as well as improving maternal bonding in mothers with preterm infants [36,37]. Our results support the hypothesis that KMM intervention reduces maternal stress, indicating that close contact and maternal touch would have a buffering effect on stress reactivity and potential impact on maternal mental health. Thus, according to other authors, KMM may be an optimal intervention to reduce anxiety and stress in mothers after preterm birth [38].

According to the literature, mothers of preterm infants admitted to the NICU are often anxious and prone to anxiety towards their babies due to the early and continuous separation of mother and child after birth [39,40]. Some studies concluded that factors, such as younger maternal age, were associated with an increased risk of postpartum depression [41,42]. However, we have not found a correlation between these two aspects. Our findings show that the kangaroo method could reduce this anxiety by allowing mothers to get close to their babies and ensure information about their infant’s condition. When mothers receive skin-to-skin contact, they focus more on their babies and make more effort to understand and respond to their babies’ behavior, which helps them build a deeper relationship between the two [43]. This behavior may lead to a rational explanation for how this mother–infant relationship or bonding may reduce anxiety [44]. On the other hand, previous research has shown that the support provided by health professionals to mothers is critical. This support guides their adaptation to their new role, makes them feel secure and confident, eliminates self-blame and feelings of inferiority, increases their sensitivity in interpersonal relationships, and gives them security [45,46].

Another important finding of our study was a greater decrease in perceived stress among mothers in the kangaroo group after the 12 days of intervention. However, we did not find statistically significant differences. Similar results were found in the study by Coşkun et al., where mothers who performed KMM had lower levels on the PSS: NICU scale and the differences in total scale scores and subdimensions in the kangaroo care group were significant [47]. The authors explain this decrease in stress levels because mothers have more direct contact with their babies. They also adapt better to the NICU environment because they communicate more with the healthcare professionals in this unit and feel more confident in performing the kangaroo method by watching their babies more closely.

Samra et al. [38] conducted a longitudinal randomized controlled trial of two groups of 40 mother–baby dyads recruited from a neonatal intensive care unit. They aimed to study the perception of stress between mothers who provided skin-to-skin contact to their late preterm infants and mothers who provided blankets. They found that mothers who provide skin-to-skin contact may experience more stress related to a more facilitated progression in the mother-baby relationship. However, other authors, such as Miles et al., concluded that skin-to-skin contact between mother and baby after extremely preterm birth produces neither benefits nor adverse consequences [48]. On the other hand, a study also found that prolonged periods of KMC could be an exhausting experience for mothers that can cause sleep deprivation and fatigue [49].

Our research shows that the average anxiety score of the intervention group was lower than the control group after the time, although it was not significant. This is one of the most relevant findings of this study. Most literature studies describe that using the kangaroo method benefits preterm newborns and their mothers [50]. However, studies have yet to be conducted in the context of the COVID-19 pandemic. Our research was performed in this context, and our findings, although showing an improvement, were not significant. A plausible explanation for this important finding is that the current study used questionnaires to assess mothers for the first time in the context of the COVID-19 pandemic. In this context, the objective parameters found significant differences in this cohort [26]. However, the responses to the questionnaires, which are subjective, although they found differences, were not significant. The most plausible explanation is that, due to the restriction and isolation measures imposed during the pandemic, the mothers’ perception of physical contact with their preterm newborns was not as positive due to the fear of being able to transmit COVID-19. This finding is one of the most relevant aspects of the present study, as future studies can compare their findings with ours.

A substantial body of scientific evidence demonstrates the positive effect of sensitive, responsive, and responsive maternal behavior on infant development, especially in premature infants. These effects may be because the protective effect of positive maternal interactive behavior is relevant: higher levels of maternal responsiveness, sensitivity, and positive mood are significantly associated with higher levels of child cognitive and social development [5,51]. Maternal role disruption is the most significant stressor, followed by infant emergence, behavior, and environmental stressors in the neonatal intensive care unit. These aspects indicate the need for intervention and counseling focused on the role of mothers, their involvement in infant care, and thus the introduction of family-centered care.

This research has some limitations. We investigated the influence of the kangaroo mother method on the occurrence of stress and sources of perceived stress related to the NICU environment. Maternal role development and sensitivity to the infant and maternal affective responses to perceived stress, such as anxiety, may be more sensitive to KMM intervention than stress occurrence scores, which only indicate whether something was perceived as stressful or not. Future studies comparing other measures of stress are needed. The mother determined the timing and duration of skin-to-skin contact sessions. The focus of the study was on the need to avoid the separation of mothers and infants. Future studies are needed to assess the impact of skin-to-skin contact on the frequency and patterns of visits. In addition, we only assessed stress at two different time points. We have not analyzed the role of fathers, as other authors have reported higher stress levels in mothers than fathers [52]. Mothers often play the role of primary caregiver and tend to spend more time in the NICU than fathers, and breastfeeding constraints are more distressing for mothers than their partners. However, it could be interesting to analyze the fathers’ responses for future research. Certain potential confounders were not considered, such as medical history, the mother’s mental health status, or the neonate’s condition at birth. These limitations should be considered when assessing the external validity of our findings. Future research is needed to assess mothers’ stress over time and should include fathers to determine whether sex differences exist in response to skin-to-skin contact. Research is also needed to explore differences in NICU designs and practices that may have contributed to the results and inconsistencies in the literature.

## 5. Conclusions

This study provides essential information for healthcare professionals caring for preterm infants in the NICU. Many parents find it challenging to adapt to the stressful environment of the NICU. Increased stress and postnatal depression in mothers are risk factors for possible less-than-favorable outcomes for preterm infants. Our results reflect that the mother kangaroo method performed for more than ninety minutes daily in the first two weeks of preterm infants may regulate stress and maternal anxiety in the early postpartum period. However, contrary to research based on biological and physiological parameters in newborns or performed before the COVID-19 pandemic, the differences found in applying subjective scales in mothers in the context of the COVID-19 pandemic were not significant. Therefore, mothers’ perception of physical contact with their preterm infants may not have been as positive due to their fear of transmitting covid. These findings have critical value for current clinical practice and can be used to develop and compare future research.

## Figures and Tables

**Table 1:** Maternal and infant demographic and clinical data of the intervention group and the control group.

Variable		Intervention Group (*n* = 56) *n* (%), Mean ± SD	Control Group (*n* = 56) *n* (%), Mean ± SD	*p*-Value *
Mothers’ variables				
Mothers’ age (years)		32.8 ± 5.6	33.2 ± 5.3	0.7 _b_
Previous pregnancies		1.6 ± 1.5	1.1 ± 1.3	0.3 _b_
Previous abortions		0.6 ± 1.0	0.6 ± 0.9	0.6 _b_
Multiple births	No	38 (33.9)	36 (32.1)	0.7 _a_
	Yes	18 (16.1)	20 (8.9)	
Smoker	No	47 (42.0)	48 (42.9)	0.8 _a_
	Yes	6 (5.4)	5 (4.5)	
Infants’ variables				
Gender	Male	28 (25)	36 (32.1)	0.1 _a_
	Female	28 (25)	20 (17.9)	
Gestational age (weeks)		31.1 ± 1.8	30.38 ± 2.0	0.1 _a_
Weight at birth (g)		1408.9 ± 301.7	1327.6 ± 292.0	0.2 _a_

* a Mann-Whitney U; b Student’s *t*-Test.

**Table 2:** Effect of the KMM regarding the questionnaires applied.

PSS: NICU: Mean NICU Scores before and after Stress for KMM and Control Groups.					
		Mean ± SD	*p*-Value *	Mean ± SD	*p*-Value *
Sights and sounds	Intervention	2.8 ± 0.9	0.2 _a_	1.9 ± 0.8	0.7 _a_
	Control	2.6 ± 1.0		1.9 ± 0.9	
Baby’s behavior and appearance	Intervention	2.4 ± 1.1	0.1 _a_	1.4 ± 0.8	0.9 _a_
	Control	2.1 ± 1.0		1.4 ± 0.8	
Relationship with the baby and her role as a mother	Intervention	2.9 ± 1.1	0.9 _a_	2.1 ± 1.1	0.6 _a_
	Control	2.9 ± 0.9		2.3 ± 01.0	
Overall score	Intervention	2.7 ± 0.8	0.5 _a_	1.8 ± 0.8	0.7 _a_
	Control	2.5 ± 0.9		1.9 ± 0.7	
State-Trait Anxiety Inventory, STAI S/T					
STAI State	Intervention	54.5 ± 3.8	1.0 _a_	51.4 ± 4.1	0.6 _a_
	Control	54.8 ± 3.2		52.0 ± 3.9	
STAI Trait	Intervention	46.7 ± 4.0	1.0 _a_	44.3 ± 6.4	0.3 _a_
	Control	46.9 ± 5.1		44.4 ± 4.2	

* a Mann-Whitney U.

## Data Availability

Not applicable.
